# Engagement of Language and Domain General Networks during Word Monitoring in a Native and Unknown Language

**DOI:** 10.3390/brainsci11081063

**Published:** 2021-08-13

**Authors:** Kelly R. Cotosck, Jed A. Meltzer, Mariana P. Nucci, Katerina Lukasova, Letícia L. Mansur, Edson Amaro

**Affiliations:** 1 FNI–Functional Neuroimaging, LIM 4–Laboratório de Investigação Médica 44 (Laboratory of Medical Investigation 44), Department of Radiology, Universidade de São Paulo, São Paulo 05403-000, Brazil; mariana.nucci@hc.fm.usp.br (M.P.N.); katerinaluka@gmail.com (K.L.); eamaro@usp.br (E.A.J.); 2 Rotman Research Institute, Toronto, ON M6A 2E1, Canada; jmeltzer@research.baycrest.org; 3 Center of Mathematics, Computing and Cognition, Universidade Federal do ABC, Santo André 09210-580, Brazil

**Keywords:** fMRI, resonance magnetic imaging, auditory attention task, speech processing, language

## Abstract

Functional neuroimaging studies have highlighted the roles of three networks in processing language, all of which are typically left-lateralized: a ventral stream involved in semantics, a dorsal stream involved in phonology and speech production, and a more dorsal “multiple demand” network involved in many effortful tasks. As lateralization in all networks may be affected by life factors such as age, literacy, education, and brain pathology, we sought to develop a task paradigm with which to investigate the engagement of these networks, including manipulations to selectively emphasize semantic and phonological processing within a single task performable by almost anyone regardless of literacy status. In young healthy participants, we administered an auditory word monitoring task, in which participants had to note the occurrence of a target word within a continuous story presented in either their native language, Portuguese, or the unknown language, Japanese. Native language task performance activated ventral stream language networks, left lateralized but bilateral in the anterior temporal lobe. Unfamiliar language performance, being more difficult, activated left hemisphere dorsal stream structures and the multiple demand network bilaterally, but predominantly in the right hemisphere. These findings suggest that increased demands on phonological processing to accomplish word monitoring in the absence of semantic support may result in the bilateral recruitment of networks involved in speech perception under more challenging conditions.

## 1. Introduction

Neuroimaging investigations into human language processing have confirmed the importance of the two “classic” brain regions in which lesions are associated with language deficits: the Broca’s and Wernicke’s areas. In addition, they have demonstrated that both of these regions are part of larger connected networks. At least three major networks with relevance to language have been delineated [1,2,3,4]. Although all three appear to be somewhat lateralized, with the left hemisphere playing a larger role in supporting language function, the homologous networks in the right hemisphere may also be involved in special circumstances [5], with variability across individuals related to such factors as age [6], multilingualism [7], education [8], and response to brain damage [9].

The lateralization of function across the two brain hemispheres has been extensively studied in neuroimaging research, with particular attention paid to how lateralization changes in the process of healthy aging and disease. Several early studies showed that older adults tend to show less asymmetrical activation patterns than younger ones, with bilateral recruitment variously interpreted as pathological or compensatory [10,11]. However, the clarification of distinct networks involved in language processing has emerged more recently in the past decade, and relatively little work has examined how lateralization of function within these networks is affected by lifestyle factors.

Two networks that appear to be innately organized with clear genetic components [12,13,14] hold particular importance for core aspects of language processing and have come to be known as the ventral stream and dorsal stream for language, in analogy to the two streams (located more posteriorly) associated with distinct aspects of vision [1,2]. The ventral language stream comprises areas in the middle and inferior temporal gyri, connected by the large white matter tract known as the inferior fronto-occipital fasciculus (IFOF). The ventral stream continues into ventral portions of the inferior frontal gyrus, especially BA 45 (pars triangularis), via the fibres of the extreme capsule and uncinate fasciculus. This pathway is linked to semantic processing and speech comprehension. The dorsal stream, more linked to speech production, sound processing, and repetition, comprises much of the superior temporal lobe, parts of the inferior parietal lobe, and a more dorsal portion of the inferior frontal gyrus, namely BA 44 (pars opercularis) [1,15]. This network is largely connected by the arcuate fasciculus, which itself comprises the most inferior portion of the larger white matter tract system known as the Superior Longitudinal Fasciculus (SLF).

In addition to these two networks, considered the “core” networks for language, a third network is frequently activated in language tasks [4], especially when difficulty increases, although it is similarly linked to task difficulty in a wide variety of cognitive paradigms including working memory (verbal or spatial), attention, cognitive control, and goal-directed behavior in general. This network goes by several names in literature, including “Multiple Demand,” “Fronto-parietal control,” “Domain-general,” and others. We adopted the term “Multiple Demand” (MD) in this paper. The network includes portions of the inferior parietal lobe superior to those of the dorsal stream, as well as frontal regions superior to the inferior frontal gyrus (IFG), including the middle frontal gyrus (BA 6) and the superior frontal gyrus (BA 8). This network is heavily activated by cognitive task demands involving verbal material, but seldom responds specifically to manipulations of semantic or syntactic content [3]. In keeping with this network’s active role across a wide variety of tasks, many researchers believe that it is involved in goal-oriented attentionally demanding cognition, which may or may not involve the verbal modality in any given instance [16]. 

The circumstances influencing functional laterality in all three of these networks involved in language processing are not well understood yet. Among the most important factors that may influence laterality in the general population are age and education. While age will always be a significant source of variability, the variability of education depends heavily on time and place. Many developed countries now enjoy universal education and a very high standard level of literacy, making it increasingly impossible to study neural variation related to different educational opportunities across the population. In some developing countries, however, a relatively large part of the population is still afflicted by poverty and low access to education. In these conditions, the effects of education and age may be analyzed by comparing subgroups of individuals co-existing within the same cultural context, each with a particular combination of these factors (i.e., young and elders; low and high literacy).

The city of São Paulo, Brazil, has a significant portion of its population, particularly in the older generation, characterized as “low literacy” or “functionally illiterate,” meaning that despite possessing normal intelligence, due to insufficient educational opportunities, many of these people are unable to read and write to any large degree [17]. Although they may have basic familiarity with the alphabet and the ability to recognize and produce certain common words (including their own name), the ability to read texts for comprehension is absent. 

This environment offers the opportunity to study how the coupling influences of age and education interact to influence lateralization of language functions. For example, while illiteracy obviously affects written language processing, much evidence suggests that it also affects spoken language processing. Illiterate people have reduced abilities on tasks that require explicit awareness of and attention to phonemes, including rhyme judgments, phoneme deletion (e.g., say cat without the “c”), and nonword repetition [18,19]. Thus, it is likely that patterns of neural activation and connectivity may differ in networks related to phonological processing. In contrast, semantic processing seems to be largely normal in illiterates, suggesting that the neural networks involved in it would appear similar across education levels [20,21].

In order to study variability in the three major language networks with functional imaging, it is necessary to choose a task paradigm that optimally differentiates them. We considered many options in the literature, hoping to find a task that can be easily explained to participants, yet somewhat challenging to perform so that some cognitive effort and engagement will be required. We preferred a task that could be done continuously in a block-design, as such a design is more efficient [22], easier to analyze, and potentially easier for participants to understand. Furthermore, we wished to maximally differentiate the engagement of the dorsal and ventral language streams within the parameters of a single set of task instructions, again to make it easy for participants to understand. 

One attractive task from the psycholinguistics literature with the right properties is “word monitoring.” In such a task, the participant listens to a continuous stream of speech, and they are requested to respond with a button press every time they hear a particular target word [23]. Word monitoring requires constant attention and vigilance, and although understanding the speech content certainly makes it easier (due to the role of prediction), one can, in principle, perform word monitoring successfully in an entirely unknown language. Therefore, we decided to contrast word monitoring in one’s native language with doing the same task in an unfamiliar language. We reasoned that performing this task in the native language should selectively engage brain regions involved in processing the semantic content of the speech signal, especially the ventral stream. Performing the same task in an unfamiliar language should require a higher level of phonological processing engagement, as well as domain general resources, as participants will entirely lack the benefit of semantic prediction and will have to rely exclusively on their ability to monitor the acoustic content of the signal. We therefore expected this task to selectively engage the dorsal stream as well as the MD network. Moreover, we have also considered the confounding effects of motor (button pressing) and simple sound processing by adding a baseline auditory and motor control condition in which subjects had to press a button when a particular tone was detected. Acoustic noise from the MR scanner during image acquisition was minimized by the use of a soft tone sequence. 

Although the predictions for selective engagement of the dorsal and ventral networks were straightforward in this study, we had less specific predictions regarding the lateralization pattern. All three networks are expected to be left-lateralized for processing verbal information, but right hemisphere engagement is frequently observed when task demands become more difficult. This study in young, healthy, and well-educated people (the typical “neuroimaging” population) provides a baseline of selective engagement for semantic and phonological processing within a single task, against which we can compare populations varying in age and education in future studies. 

## 2. Materials and Methods

### 2.1. Participants

This study was approved by the Research Ethics Committee of the *Hospital das Clínicas, Faculdade de Medicina* (Medical School) from the University of São Paulo (HC-FMUSP) (Protocol No. 150/14, 26 May 2014), and all participants gave their formal consent to participate in this study through the “Free and Clarified Consent Term”.

### 2.2. Inclusion or Exclusion Criteria

Inclusion: The participants of this study were young adults, aged from 21 to 35 years-old, healthy, right-handed, monolingual (no prior acquisition of a second language before the age of 10), with more than 15 years of formal schooling, living in the city of São Paulo for at least 8 years, with no history of neurological or psychiatric illnesses. They had normal or corrected-to-normal vision.

Exclusion: We excluded volunteers who were not eligible for MRI. Some participants were also included for reasons such as: artifacts shown on images, technical issues during the performance of the task, or accuracy scores below 70% in the word monitoring task.

### 2.3. Word Monitoring Task

The experimental paradigm was a block design, with each block lasting 30 s, alternating between 3 different conditions (Native Language: NL, Unknown Language: UL, Baseline: BL), and totaling 20 blocks. Two active conditions (conditions NL and UL) were interleaved with the baseline condition (BL).

Condition NL consisted of short audio excerpts of a single text written in the native language, and condition UL was in the same way made of excerpts of a text in the unknown language. The baseline condition BL was formed by silence punctuated by occasional presentation of a simple sinusoidal tone (f = 1056 Hz, peak value = 0.5 dB, and rise/fall times equal to 0.038 s), each one with a duration of 1 s. The baseline condition aimed at controlling the general context of vigilance for an occasional auditory stimulus, auditory response to acoustic stimuli per se, and the voluntary motor response.

**Figure 1:**
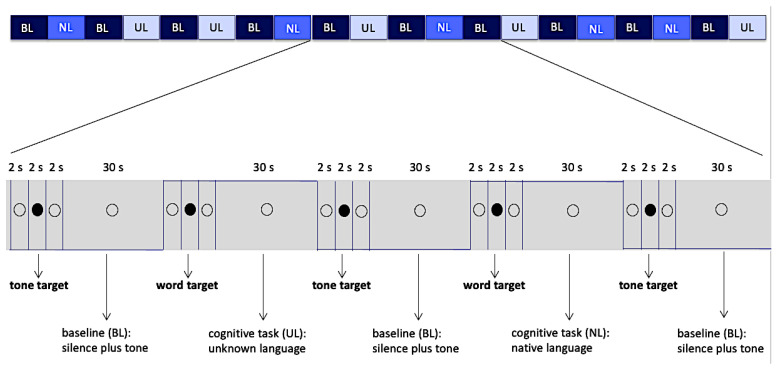
Block design, 20 blocks of 30 s and intervals of 6 s between them. The empty circle and filled circle were visible to the volunteers while they listened the audio. Abbreviations: BL—Baseline, NL—Native Language, UL—Unknown Language.

Conditions NL and UL were presented in a pseudorandom sequence, and each condition was preceded by a word that was a target for conditions NL and UL or simply a sinusoidal tone, which was the target for the baseline (Figure 1). The volunteer was instructed to respond to these targets by pressing a single button (Zurc & Zurc, São Paulo, Brazil), which was held in their left (non-dominant) hand during the whole scanning procedure. Although the total number of words in the blocks of conditions NL and UL were different, the amount of targets in the blocks was matched, and this amount varied from 1 to 4 target words per block. In order to balance the motor response, the amount of sinusoidal tones in each block in the baseline condition also varied from 1 to 4. The target word in each language (native and unknown) was always the same for all blocks within that condition and had the same meaning in both languages (water: agua in Portuguese, mizu in Japanese). 

Active conditions (NL, UL) totaled 10 blocks, 5 blocks of each condition, while the baseline condition (BL) totaled 10 blocks. Each block, in all three conditions, was preceded by intervals of 6 s in duration. These intervals were divided as follows: 2 s of presentation of the hollow black circle, with no associated audio; followed by 2 s of presentation of a full black circle associated with audios of the target words (when they preceded the active conditions) or sinusoidal target tone (when they preceded a baseline condition); and finally, another 2 s presentation of the hollow black circle, with no associated audio (Figure 1). The full black circles preceded the stimuli (target words or target tone) presentation and cued the volunteer to note them. Total duration of data collection was 12 min and 10 s. Auditory stimuli and images projected on screen were generated through e-Prime software (Psychology Software Tools version 1.1, Pittsburgh, PA, USA). This software also collected behavioral data related to the motor response.

#### 2.3.1. Texts, Audios and Sound Systems Used in the Paradigm

The text excerpts used for auditory stimuli in NL and UL conditions were equated according to the semantic content and vocabulary, consisting of a children’s story translated into both languages. Both versions were matched according to semantic and lexical factors, although participants did not understand the unknown language. The audio stimuli were recorded in a professional studio (Universidade Braz Cubas, Mogi das Cruzes, Brazil) by a bilingual female fluent from early childhood in both languages, who currently works as an early childhood educator for the Japanese Language in São Paulo. The audio was edited for volume, time, and tone using Audacity software (version 2.1.3 (Audacity Team, Pittsburgh, PA, USA)), fixing each audio excerpt to a length of 30 s. 

For validation of the paradigm, pilot trials were carried out with 6 volunteers with two different speakers for the audio stimuli, using the same functional magnetic resonance equipment used in the main experiment. The data from these 6 volunteers were not used for the final sample of this study.

The audio signals were transmitted to subjects through amplification systems, transducers and headphones, with the latter featuring high noise reduction, as well as through speakers installed on the panel (equipment built and adapted for the magnetic resonance environment (Zurc & Zurc, São Paulo, Brazil)).

#### 2.3.2. Positioning and Orientation in the Scanner

Effects of eye movements were minimized by instructing participants to listen attentively through the headphones while keeping eyes open and focused on a circle shown in the center of screen. Head movements were reduced by using lateral cushions and an inelastic tape was gently placed in subjects’ forehead in order to provide sensory feedback in case involuntary movement occurred, allowing self-correction and return to original head position.

#### 2.3.3. Pre- and Post-fMRI

Prior to entering the scanner, all participants performed a quick practice in a task analogous to the paradigm’s by making use of a portable computer located in a dedicated testing room. For this training, 3 blocks for each active condition (NL and UL) were used, each preceded by one block of the baseline condition. Although the experimental design of the training was analogous to that of the experiment, based on the same text, the excerpts used in the training, as well as the target words, were different from those presented during the experiment.

The duration of the training varied among the volunteers (between 8 and 20 min). Since there was an exhibition of stimuli for the training, we maintained the same order of exhibition, keeping a pattern for both the instructions and procedures and for this mentioned exhibition, during the training. All the trainings, as well as the entire experiment, were carried out by the same researcher. All the volunteers reached 95% of correct presses in the training.

#### 2.3.4. Image Acquisition

MRI images were collected by a 3 Tesla MR system (Philips Achieva 3T, Eindhoven, Netherlands), equipped with 80 mT/m gradients and an 8-channel head coil.

Anatomical T1-weighted images, with axial acquisition, were acquired by means of the following parameters: repetition time (TR) = 7.0 ms; echo time (TE) = 3.2 ms; acquisition field-of-view (FOV) 230 × 183 × 140 mm; thickness of 60 mm; flip angle = 8 degrees.

For fMRI, volumes of the entire brain were acquired via EPI soft tone sequences with a 3.7 factor from slew rate [24], with the following acquisition parameters: TR = 2 s; TE = 30 ms; voxel volume = 3.0 × 3.0 × 4.0 mm^3^; 31 slices; gap of 0.5 mm; matrix size = 3 × 4 × 4 mm; and 365 volumes.

Axial T2-weighted images (Axial Fluid Attenuated Inversion Recovery [FLAIR]) were also collected, with TR = 11.000 ms; TE = 130 ms; FOV = 230 × 183 × 140 mm; gap = 0.5 mm; 28 slices; thickness of 4.5 mm; and voxel of 0.65 × 0.86 × 4.5 mm, aiming at assessing lesions in the white matter and occasional incidental findings.

#### 2.3.5. Image Processing

fMRI data were processed using FMRIB Software Library (FSL: version 6.0 [Center for Functional MRI of Brain, Analysis Group, Oxford, United Kingdom, http://fsl.fmrib.ox.ac.uk/fsl/fslwiki] (20 May 2020)).

Individual images (first level) were preprocessed comprising the following steps: For motion correction, data was preprocessed through MCFLIRT (Jenkinson et al., 2002); slice-timing correction was performed by means of Fourier analysis of time series (regular up); for the removal of all non-cerebral matter from the images, we used the Brain Extraction Tool (BET) [25]; we applied spatial smoothing through a Gaussian kernel of FWHM 5 mm; and a high-pass time filter for the removal of low-frequency artifacts (Gaussian weighted least squares, sigma = 100.0 s). A statistical analysis of the time series with the use of GLM (General Linear Model) was also carried out, with local autocorrelation [26], comprising two predictors for the task (NL and UL conditions) and BL condition as the baseline. The contrasts were generated for NL versus BL, UL versus BL, and NL versus UL at the individual level. Spatial registration was done by warping the participant’s high-resolution 3D T1-weighted brain image into the space defined by the template atlas MNI-152 (Montreal Neurological Institute, Montreal, QC, Canada) using a linear registration tool (FMRIB’s Linear Image Registration Tool (FLIRT)), affine with 12 degrees of freedom. Statistical group analysis was concentrated in the contras NL versus UL in both directions (NL > UL and UL > NL).

Individual contrasts and variability maps were submitted to a mixed effects model to assess generalizability to the population. The activation group maps were assigned a non-parametrical threshold using clusters to determine a z-score > 3.1 and a (corrected) cluster significance threshold *p* = 0.05 [27].

### 2.4. Behavioral Data

To evaluate behavioral performance, each of the conditions was analyzed to extract the following values: “hit rate” (HR), “hit reaction time” (HRT), and “false alarm rate” (FA). Correct responses to the target (hits) were considered as button presses that occurred after the target onset within a time window of 2.5 s. We performed an evaluation of hit percentage (correct responses) for each condition (active and baseline). The minimum HR considered acceptable (cut-off value) was a mean of 80% across blocks for the active condition NL. HRT analysis was done considering all correct responses to targets.

**Figure 2:**
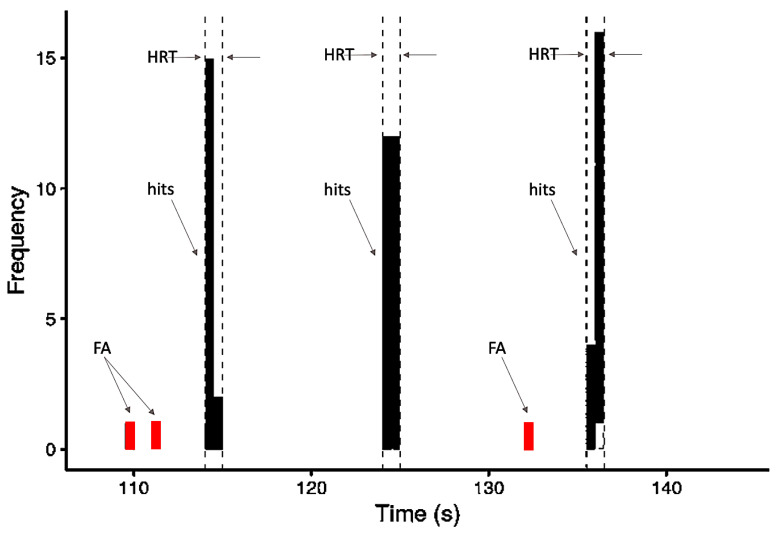
Schematic model of the frequency distribution of behavioral variables based on responses occurring within one block: hits, false alarms (FA), and the 2.5-s time window within which responses were counted as hits and the HRT (hit reaction time) calculated.

The FA was considered a button press that occurred at any time in a block outside of the 2.5 s window following each target stimulus (Figure 2). The false alarms rate (*FAR*) was calculated as the ratio of the sum of times the volunteer pressed the button improperly (false alarms) and the total number of times the button was pressed by the same volunteer (hits + false alarms), as follows:

(1) $$FAR\;\left(\%\right)=\frac{\sum^{\;}false\;alarms}{\sum^{\;}\left(hits+false\;alarms\right)}$$

In addition, we calculated D-prime scores (sensitivity measure) considering the hit rate (HR) and false alarm rate (FA) performances in all participants for each condition evaluated (NL, UL, and BL) [28].

All data are presented using the mean and standard deviation across participants. Shapiro–Wilk and Levene’s tests were used to determine normality and variance equality, respectively. Accuracy and HRT were analyzed by ANOVA test, and if significant, post-hoc tests with Bonferroni correction were performed, using the 0.05 level of significance for all the analyses. Statistical analysis was carried out in R (R: A language and environment for statistical computing. R Foundation for Statistical Computing, version 3.6.3, Vienna, Austria, Available online: http://www.r-project.org/index.html (accessed on 9 August 2021)).

## 3. Results

### 3.1. Demographic Results

Thirty-five participants volunteered for the study; however, 10 volunteers were excluded. Three had previous background knowledge of a few words in the unknown language; one presented neuropsychological results below expectations for the group average; two were left-handed; two did not finish the MR testing; one claimed after the experiment that they did not hear the words properly; and one volunteer presented a hit rate below 80% in condition 1 (native language) of the paradigm. The final sample of the study was composed of 25 young adults (16 women, average age = 26.57 ± 5.23 years old; 9 men, average age = 27.78 ± 5.19 years old) with an average schooling of 15.82 ± 1.30 years.

### 3.2. Behavioral Assessment in the Auditory Attention Task

For each of the three conditions, three analyses of the behavioral results were carried out: the hit rate (HR), the false alarms rate (*FAR*), and the hit reaction time (HRT) of correct answers.

#### 3.2.1. Accuracies and Errors

**Figure 3:**
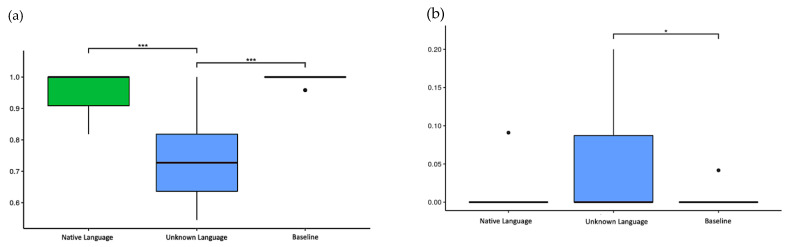
The accuracy and false alarm rate in the task. (**a**) Hit rate (HR) of three conditions: native language (0.957 ± 0.072), unknown language (0.735 ± 0.128), and baseline (0.995 ± 0.014); (**b**) False alarm rate (*FAR*) of three conditions: native language (0.004 ± 0.019), unknown language (0.038 ± 0.062), and baseline (0.002 ± 0.009). * *p*-values < 0.05 and *** *p*-values < 0.001.

The HR was 95.7% for the native language, 73.5% for the unknown language, and 99.5% for the baseline. The Kruskal–Wallis test showed a significant difference between conditions (*p* < 0.001), and the pairwise Wilcoxon test showed the unknown language elicited significantly fewer correct responses than both the native language (*p* < 0.001) and the baseline (*p* < 0.001) (Figure 3a).

Although the HR of the unknown language presented inferior outcomes in comparison to the other conditions, results reveal that the accuracy score was evenly distributed over all blocks. Consequently, the miss rates were 4.3% for the native language, 26.5% for the unknown language, and 0.5% for the baseline.

The *FAR* was 3.8% for the unknown language, 0.4% for the native language, and 0.2% for the baseline. The Kruskal–Wallis test showed significant differences between conditions (*p* = 0.008), and the pairwise Wilcoxon test only showed a statistically significant difference between the unknown language and the baseline (*p* < 0.05) (Figure 3b).

In both analyses (HR and *FAR*), the Shapiro–Wilk and Levene’s tests were applied previously, which showed that the data had a non-normal distribution (*p* < 0.001) and a non-homogeneous variance (*p* < 0.001), respectively, and a non-parametric ANOVA was adopted for this analysis.

The following D-prime scores were observed: 5.263 for NL, 3.154 for UL, and 5.985 for BL; all results were close to the best sensitivity condition reported for this score (the maximum achievable D = 4.65 in the ideal condition – HR = 1 and FA = 0) [28] (Table 1).

#### 3.2.2. Hit Reaction Time (HRT)

**Figure 4:**
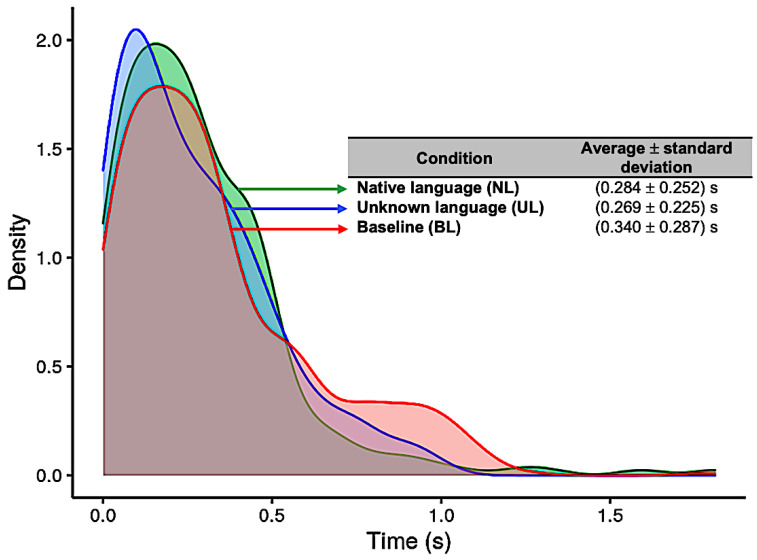
Distribution of reaction times (in seconds) for each of the three conditions: Native language (in green); Unknown language (in blue); and Baseline (in red).

The results for the distribution of the HRT in each condition (NL, UL, and BL) are presented in Figure 4. For the NL condition, the average and standard deviation was 0.28 ± 0.25 s (native language, blue curve); for the UL condition, it was 0.27 ± 0.23 s (unknown language, green curve); and for the BL condition, 0.34 ± 0.29 s (baseline, red curve) (Figure 4). The Kruskal–Wallis test showed significant difference between conditions (*p* = 0.0103), and the pairwise Wilcoxon test showed statistically significant differences between UL and BL (*p* = 0.024). Previously, the Shapiro–Wilk and Levene´s tests were applied, which showed that the data had a non-normal distribution (*p* < 0.001) and a non-homogeneous variance (*p* < 0.001), respectively, and a non-parametric ANOVA was adopted for this analysis.

### 3.3. fMRI Results

**Figure 5:**
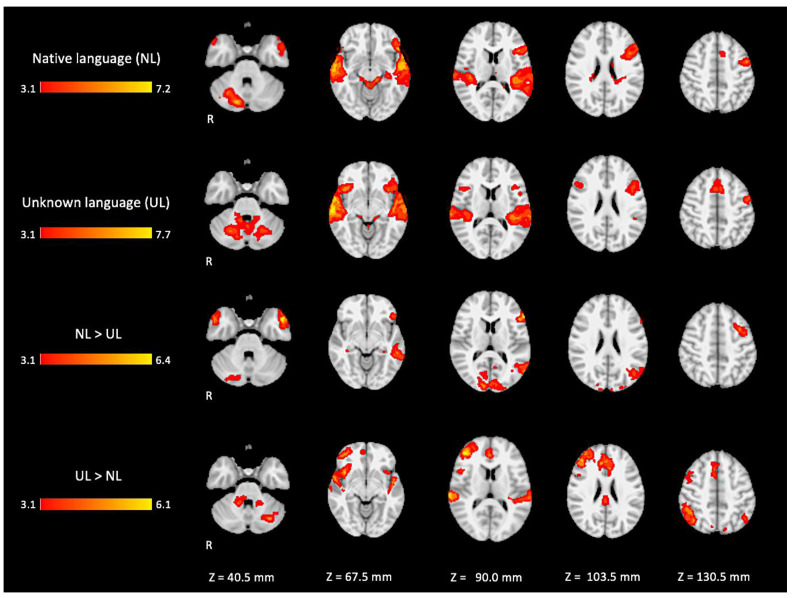
Two-dimensional fMRI map of auditory attention task (*n* = 25). BOLD signal clusters (in red) are observed in the following brain regions: First row is condition 1 (native language—NL) versus baseline; second row is condition 2 (unknown language—UL) versus baseline; third row is the contrast “native language” > “unknown language”; fourth row is the contrast “unknown language” > “native language”. (Red–yellow scale indicates the z-score > 3.1; *p* < 0.05).

**Figure A1:**
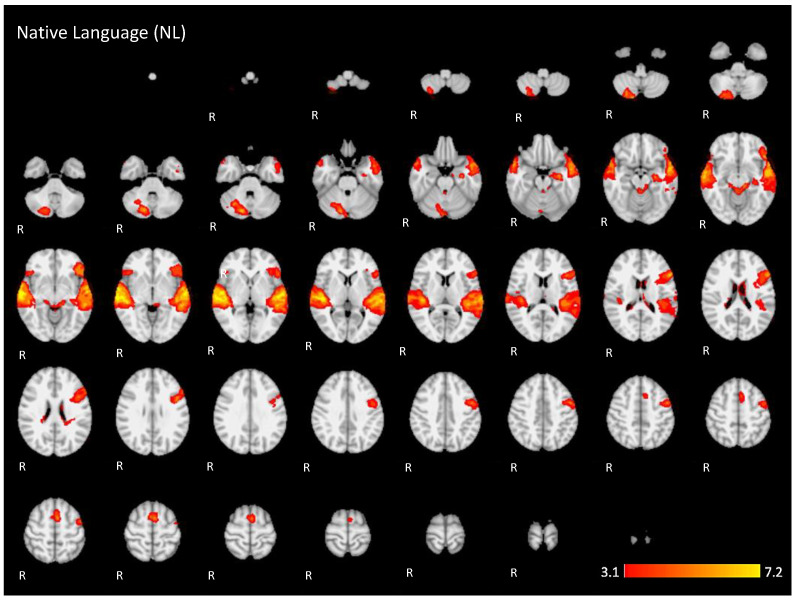
Two-dimensional fMRI map of auditory attention task (n = 25) for the contrast native language condition higher than baseline. (Red–yellow scale indicates the z-score > 3.1; *p* < 0.05).

In the active condition NL (native language) compared to the baseline, significant BOLD signal activations were observed in regions considered as part of the ventral and dorsal language networks: the right inferior frontal gyrus pars triangularis, the bilateral superior temporal gyrus with extended activation into the middle temporal gyrus, and the inferior temporal gyrus in the left hemisphere. Furthermore, activation was found in areas described as part of the Multiple Demand network in the middle frontal gyrus, the paracingulate gyrus, and the precentral gyrus. Additional activation was found in the right posterior lobe of the cerebellum and the bilateral middle parahippocampal gyrus, with extension into the left hippocampus, as shown in Figure 5 (images of first line) and Figure A1 (Appendix A).

**Figure A2:**
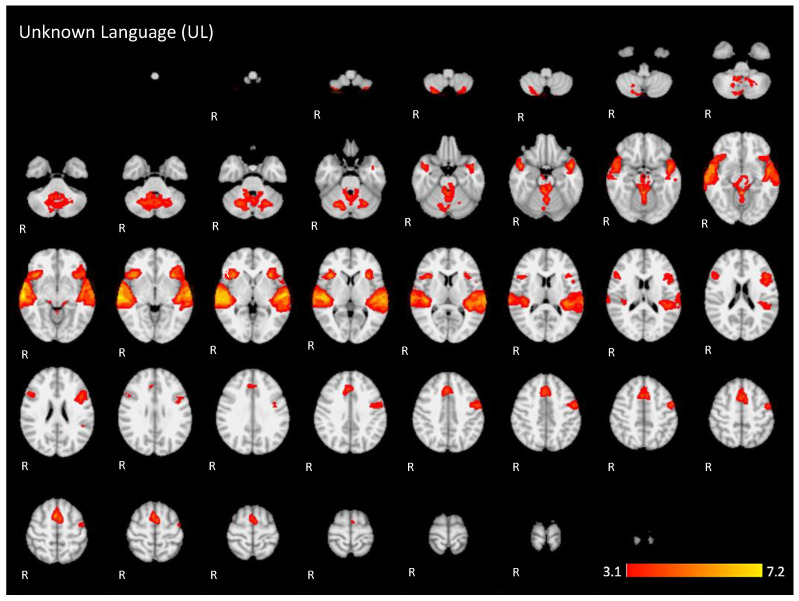
Two-dimensional fMRI map of auditory attention task (n = 25) for the contrast unknown language (UL) condition higher than baseline condition. (Red–yellow scale indicates the z-score > 3.1; *p* < 0.05).

For the active condition UL (unknown language) compared to the baseline, significant activations were observed in similar brain regions activated and described in condition NL, comprising the ventral and dorsal language networks, however, with more activation in the right hemisphere. In addition, relatively more activation was found in regions of the dorsal language network such as the bilateral inferior frontal gyrus, the pars opercularis, and the superior temporal gyrus, as shown in Figure 5 (images of second line) and Figure A2 (Appendix A).

**Figure A3:**
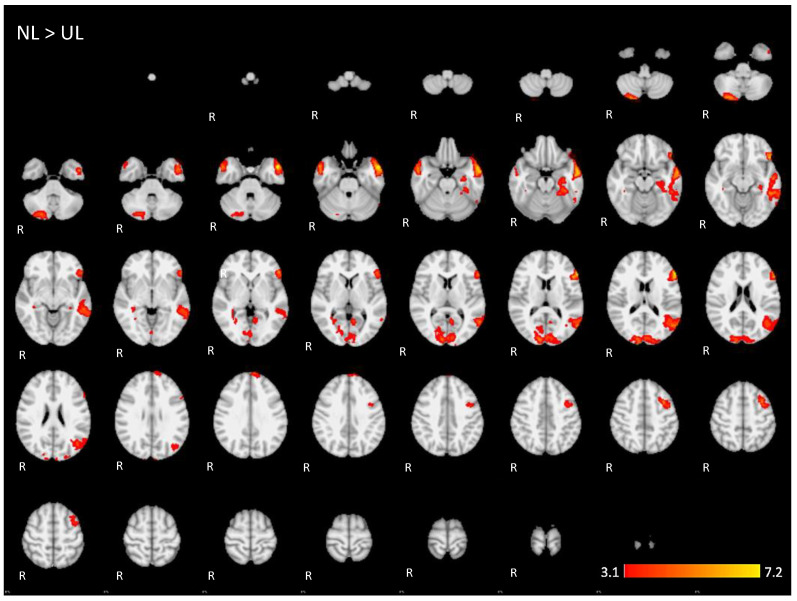
Two-dimensional fMRI map of auditory attention task (n = 25) for the contrast native language (NL) condition higher than unknown language (UL) condition. (Red–yellow scale indicates the z-score > 3.1; *p* < 0.05).

When directly comparing both active conditions, in the contrast (NL > UL), the ventral network was especially enhanced with significant left hemisphere activations covering regions from the temporo-occipital junction into the middle temporal gyrus and on into the temporal pole and the inferior frontal gyrus, mainly in the pars triangularis. The right temporal pole was also activated. Additionally, the bilateral occipital lobe, cuneus, and the posterior lobe of right cerebellum were also activated (images of third line of Figure 5 and Appendix A Figure A3).

**Figure A4:**
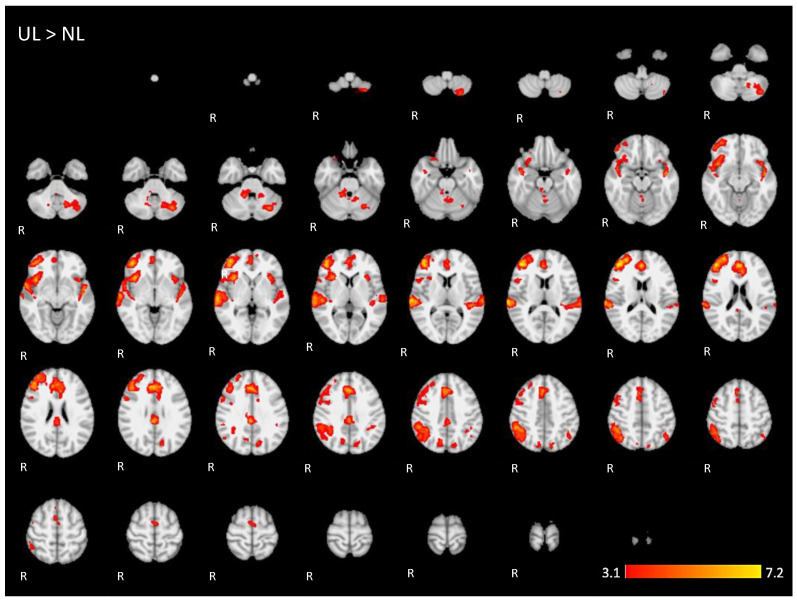
Two-dimensonal fMRI map of auditory attention task (n = 25) for the contrast unknown language (UL) condition higher than native language (NL) condition. (Red–yellow scale indicates the z-score > 3.1; *p* < 0.05).

For the contrast (UL > NL), activation was found in additional regions from the dorsal language network and multiple demand networks, such as the angular and supra-marginal gyrus in the bilateral inferior parietal cortex, the bilateral paracingulate and cingulate gyrus, and the bilateral insular cortex (images of fourth line of Figure 5 and Appendix A Figure A4).

The brain activation maps are shown in Figure 5 using the main slices of the 2D fMRI maps for the active conditions NL and UL and for the contrasts NL > UL and UL > NL. The 2D fMRI maps of all brain slices for both active conditions (NL, UL) and for both contrasts (NL > UL and UL > NL) are in Appendix A (Figure A1, Figure A2, Figure A3 and Figure A4, respectively).

**Figure 6:**
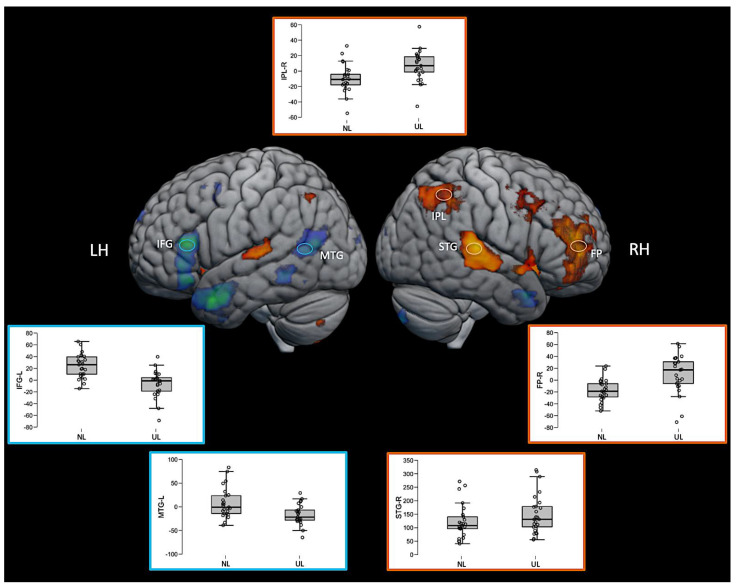
Three-dimensional fMRI map during the auditory attention task. The brain tridimensional rendered images represents the regions activated when comparing NL > UL contrast (red–yellow color scale indicates the z-score) and the regions activated when comparing UL > NL contrast (blue–green color scale indicates the z-score). The boxplots represents the percent change in beta values (y-axis) of each anatomical region of interest (IFG—inferior frontal gyrus; MTG—middle temporal gyrus; IPL—inferior parietal lobule; STG—superior temporal gyrus; FP—frontal polo; LH—left hemisphere; RH—right hemisphere), and in the x-axis the conditions NL and UL are identified. For representation purposes, the ventral stream network regions are depicted in blue (brain activation clusters and boxplot’s edges) and dorsal stream network regions in orange (brain activation clusters and boxplot’s edges), illustrating the relationship between task contrasts and brain networks.

Interestingly, some regions such as the right inferior parietal lobe (IPL) and right frontal pole (FP) were significantly activated in this direct contrast of the two active language conditions, but not for either language condition compared with the baseline. The inspection of the average fMRI signal change (beta values) in these regions revealed that the region was positively activated relative to the baseline for the unknown language and negatively activated for the native language, but neither of those comparisons to the baseline was sufficiently large to achieve statistical significance. However, the difference between them directly was significant. The pattern can be seen in the box plots in Figure 6. The 3D fMRI maps of Figure 6 showed only the contrasts NL > UL and UL > NL, highlighting the pattern of activation of the main brain regions involved in the ventral (IFG and MTG in blue hoops and boxplot’s edges of Figure 6) and dorsal (IPL, STG, and FP in orange hoops and boxplot’s edges of Figure 6) language streams. The percent signal change in the beta values of the corresponding brain regions are represented in the boxplot insights in the same figure.

Table 2 presents activated clusters, the MNI 152 coordinates of its activation peaks and respective locations are shown in Figure 5 and Figure 6.

## 4. Discussion

This study employed an auditory word monitoring task in which participants had to listen actively to a continuous stream of speech to detect a target word. Word monitoring tasks have been used extensively in psycholinguistics research to study aspects of speech comprehension, including the role of syntactic, lexical, phonological, and pragmatic aspects of the speech being presented [23]. These manipulations assume that the listener understands the speech—indeed, performing a monitoring task does not seem to impede comprehension despite the added cognitive demand [29]. Word monitoring is typically performed with high accuracy, as seen in our data with 95.7% correct in the native language condition.

Although word monitoring in one’s native language is an extensively studied task, we are not aware of any previous study (behavioral or neuroimaging) requiring participants to perform it in an unknown language. Without comprehension of the speech signal, few experimental manipulations are available within the context of the task, but comparing it with monitoring in the native language offers a straightforward way to examine the relative engagement of distinct networks for semantic and phonological aspects of the task, with the unknown language expected to place increased demands on phonological processing, short-term memory, and auditory attention. We expected participants to perform well above chance in the unknown language but to find it more challenging. The behavioural data confirm that the task is more difficult, with a hit rate of 73.5%, indicating that participants missed the target word fairly often. On the other hand, when they did detect it successfully, it was no more difficult to respond to, with equivalent response times in both languages. Participants did have longer response times in the baseline tone detection task, however, despite very high accuracy (99.5%). Inspection of the RT distribution (Figure 4) shows that this difference is driven largely by more trials with abnormally long RTs in that condition (i.e., a fat tailed distribution), suggesting that participants sometimes had attention lapses within this relatively undemanding task.

The present study was designed to provide a basis for studying variability in language network recruitment and lateralization associated with age, education, and literacy. Because learning to read strongly improves phonological processing skills even with entirely audio material [19], we reasoned that a task manipulation with increased demands on phonological processing, and decreased reliance on semantic comprehension, could be ideal for revealing differential engagement of language networks across populations varying in these characteristics. To control for certain task aspects common to both conditions, we contrasted both additionally with a tone-detection task involving similar demands for sustained vigilance and occasional motor responses to a target.

The comparison of our findings with previous studies is limited in that no previous study has used the same manipulation, but the selective activation seen for both contrasts can be compared to other studies that have sought to isolate semantic and phonological processing. We found that the native language condition, compared to the unknown language, selectively activated left-lateralized regions classically associated with speech comprehension and with lexico-semantic processes, including MTG [13], posterior STG (Wernicke’s area), and IFG (Broca’s area). Additionally, this condition activated the most anterior portions of the temporal lobe (temporal pole) bilaterally. The anterior temporal cortex has been identified as a critically important region for high-level amodal aspects of semantic processing and comprehension, with representation in both hemispheres [30,31,32], consistent with our finding of selective activation for comprehended vs. non-comprehended speech bilaterally in this region. One early PET study [33] also compared listening to stories in a native (French) and unknown (Tamil) language, along with other conditions, although it only examined passive listening with no behavioural response required. That study showed similar findings, with the native language selectively activating the left IFG, MTG, STG, and bilateral temporal poles, while the unknown language only activated the bilateral auditory cortex in the STG. However, the same study did not show any selective activation for the unknown language, likely reflecting the lack of behavioural demands. Overall, these findings illustrate that the contrast (areas more active in native language compared to an unknown language) within the common task framework of word monitoring is a simple and effective way of isolating activation throughout a network of brain regions involved in semantic processing [34].

The reverse contrast (areas more active in unknown language compared to native language) produced a more extensive set of activations, largely in areas outside the classical left-lateralized language networks. In the left hemisphere, increased activation was observed in the superior temporal gyrus (STG), part of the dorsal stream of speech processing [1], and an area heavily linked to auditory and phonological processing in general. Notably, this activation is bilateral, and the bilateral STG was also strongly activated in both language conditions relative to the tone-detection baseline. The bilateral STG activation suggests that, although the raw level of audio input is equivalent in both conditions, participants may be engaging in more intensive auditory analysis in order to complete the word monitoring task in the complete absence of semantic support.

Relative to the native language, word monitoring in the unknown language also produced activation in the anterior insula bilaterally. This region, especially on the left, is also associated with dorsal-stream speech processing, both in production and comprehension [35]. Thus, looking within regions linked to the dorsal and ventral language networks, our results point to a dissociation between them, with native-language processing preferentially activating the ventral stream networks involved in semantics and unknown-language processing activating certain dorsal stream regions more closely associated with phonological processing and speech production. This dissociation is incomplete, however, in that some portions of the dorsal stream were also preferentially activated for native-language processing. For example, almost the entire left inferior frontal gyrus (LIFG) was activated selectively by native-language processing, comprising both ventral portions (BA 45) linked to semantic processing, as well as dorsal portions (BA 44) linked to phonology. Intriguingly, another area presented a dissociation between hemispheres. Activation in the left premotor cortex, along the anterior bank of the precentral gyrus and extending anteriorly into the superior frontal gyrus, was selectively increased for the native language, but its right hemisphere homolog exhibited increased activation for the unknown language instead.

Beyond the two core language networks, word monitoring in the unknown language resulted in greater activation in a number of fronto-parietal regions that are frequently activated under conditions of greater cognitive challenge—the Multiple Demand (MD) network. This included a portion of the left inferior parietal lobe, but surprisingly, the majority of the selective activation for the unknown language was in the right hemisphere. Activated regions within this network included the inferior parietal lobe, the premotor cortex, and the anterior cingulate cortex. Interestingly, several of these regions did not exhibit significant activation in the word monitoring tasks alone when compared to the tone-monitoring baseline. Inspection of signal changes revealed that these regions tended to activate positively compared to the baseline during unknown language processing but tended to deactivate (negative BOLD change) during native language processing, resulting in a significant difference when the two conditions were directly compared. Notably, along with extensive recruitment of the right hemisphere MD regions, the unknown language condition also activated the left cerebellum, which is preferentially connected to the right hemisphere of the cortex and frequently shows co-activation with it [36].

Activation of the MD network is unsurprising given the increased demands on attention and phonological working memory, the latter being necessary in order to make decisions on auditory material heard in the past few seconds while continuing to monitor the input. However, the pattern of right-dominant lateralization is somewhat surprising. Since the word monitoring task, despite the total lack of comprehension, involves close attention to phonetic content, one might still expect greater engagement in areas normally responsible for phonological aspects of normal language processing, which are also thought to be left-lateralized [1]. It is informative to compare our results to three previous lines of research that have sought to delineate brain regions involved in extracting information from speech signals: effortful listening to degraded speech, short-term memory for the speech of varying degrees of meaningfulness, and engagement of networks during naturalistic speech comprehension. We consider each in turn, illustrating how our findings point to an intriguing hemispheric dissociation not previously observed so directly.

Several studies have applied digital processing to speech to degrade its intelligibility, forcing participants to engage in “effortful listening” to extract the meaning. Of note, [37] found that activation in the bilateral temporal cortex was positively correlated with speech intelligibility in regions overlapping with those responding preferentially to the meaningful native language condition in our study. Increased signals to distorted speech, reflecting increased effort to extract meaning, were seen in more dorsal regions but only in the left hemisphere. A follow-up study [38] found that bilateral STG and IFG were more responsive when participants were attending to a speech stimulus vs. a visual or auditory distractor, and also saw increased activity to distorted speech when it was attended to in bilateral inferior parietal and insular regions, which were selective for the unknown language in our study. This suggests that some areas selective for the unknown language in our study are involved in directing attention to challenging speech stimuli, but it does not clarify whether this increased activity represents semantic or phonological processes.

Other studies have explored the role of meaningfulness by requiring participants to engage in short-term memory maintenance of verbal material, which may be highly meaningful (e.g., sentences) or less meaningful (lists of words, or lists of nonwords). In a detailed review of neural activity involved in verbal short-term memory, [4] delineates the three networks discussed here and suggests that the left-lateralized ventral and dorsal streams are involved in semantic and phonological aspects of linguistic item maintenance, respectively. Meanwhile, the MD network is recruited bilaterally for the deployment of attention involved in the maintenance of serial order for the successful completion of tasks such as a sentence or list repetition, with increasing involvement of these networks as semantic support decreases (e.g., nonword lists > word lists > sentences). Majerus [39] proposes that a region centered on the right interparietal sulcus may play a particularly important role in the deployment of attention across many domains of cognition, including numerical cognition [40,41], whereas its left hemisphere counterpart may be more specifically involved in language due to its connections to the dorsal and ventral language networks. Our results are consistent with this, with this region activated in both hemispheres, but preferentially on the right in the absence of any comprehension of the unknown language. One should note, however, that the word-monitoring task does not explicitly require processing of serial order. However, it does require verbal short-term memory, with participants having to decide if they heard the target word in the past few seconds while continuing to take in new auditory input. This ability could be subserved by similar neural resources such as those involved in the verbal rehearsal underlying repetition of non-meaningful material.

In sum, the activation for the unknown language relative to the native language monitoring is associated with differences in phonological, lexical, syntactic, and semantic processing, as well as to the processing of the different prosodic features of Japanese. fMRI studies on prosody found right-literalized activation in the superior temporal, dorsolateral, and medial frontal, insular/fronto-opercular cortex, and cerebellum [42].

Finally, several recent studies have specifically attempted to compare the sensitivity to language-related variables of the core dorsal and ventral language networks with the more dorsal multiple demand network in the comprehension of naturalistic speech. A meta-analysis of data from several experiments showed that the MD network responds more strongly in story comprehension when there is an explicit task, as in our word monitoring paradigm, and responds more strongly to word lists than sentences [43]. Activity in the language networks is more similar across multiple participants listening to the same story than the activity in the MD network is [3], and the language networks more closely track word by word variation in processing difficulty, as predicted by psycholinguistic variables and empirical measures such as self-paced reading time [44]. These findings are consistent with our clear dissociation of left-hemisphere language networks that are more responsive to the native language and domain general regions in both hemispheres (but especially the right) responding more to the unknown language.

Although the engagement of the MD network to support a mentally challenging, attention-demanding task such as word monitoring in a foreign language is not especially surprising given the role of this network in diverse aspects of cognition, the strong right lateralization of the selective activity was a surprise to us. Studies of language comprehension and repetition have sometimes found selective engagement of the right hemisphere MD network for linguistic tasks with higher difficulty levels, such as repeating lists of words or nonwords without sentence structure [39,45,46], generating words in a fluency task with added time pressure from external pacing [47], and re-analyzing sentences with high syntactic complexity after they have already been heard [48]. The selective activation of the right inferior frontal gyrus for the unknown language in our study, in the vicinity of the Broca’s area homolog but with a more anterior distribution impinging into dorsolateral PFC and frontal pole (e.g., BA 9/46), is also consistent with studies that have observed the activation of this area in speech production tasks requiring more difficult selection of words to produce [49,50].

Our study has some limitations that should be considered when interpreting the results. First, the male/female ratio is unbalanced. Although in our design the comparisons were performed between conditions in the same subject, the differences found may be affected by gender differences in language processing. Studies focusing specifically in this subject are warranted.

## 5. Conclusions

In summary, our results suggest that participants attempting the word monitoring task in an unknown language, thus without any aid from semantic processing and prediction, rely increasingly on right-lateralized domain-general networks to support sustained attention and working memory for detecting a phonological target. This may involve some of the same mechanisms leading to increased right hemisphere engagement when healthy participants process more challenging language input and which may also support partial recovery in patients with aphasia due to left-hemisphere damage [51,52,53]. The simple comparison of the same word monitoring task in a native and an unknown language may thus prove to be a practical and effective tool for monitoring the recruitment of such resources in different populations, including tracking the effects of aging, education, and literacy on the brain’s organization for language.

## Figures and Tables

**Table 1:** Accuracy of behavioral data (HR and FA) as a function of conditions.

Condition	HR (Sum)	FA (Sum)	D-Prime
Native Language (NL)	22.011	0.092	5.263
Unknown Language (UL)	16.905	0.874	3.154
Baseline (BL)	22.885	0.046	5.985

Abbreviations: HR—hit rate; FA—false alarm rate.

**Table 2:** Description of the clusters of the group statistical maps presented in Figure 5 and Figure 6.

	Z Score	MNI Coordinates (mm)			Brain Regions
		x	y	z	
NL	7.14	−58	−16	0	Left superior temporal gyrus
	7.24	62	−14	−8	Right middle temporal gyrus
	6.18	16	−78	−30	Posterior lobe of right cerebellum
	5.6	−4	4	58	Left supplementary motor cortex
	5.25	0	−40	−14	Anterior lobe of cerebellum
UL	7.26	−58	−16	0	Left superior temporal gyrus
	7.7	66	−20	−4	Right superior temporal gyrus
	6.04	30	−72	−60	Right cerebellum
	5.96	4	10	56	Right Superior frontal gyrus/premotor cortex
	4.55	−30	−72	−58	Left cerebellum
NL > UL	6.47	−58	28	14	Left inferior frontal gyrus, *pars triangularis*
	4.93	14	−92	14	Right occipital lobe/cuneus
	4.72	−56	−70	14	Left middle temporal gyrus
	5.01	26	−82	−46	Posterior lobe of right cerebellum
	4.87	−36	8	48	Left middle frontal gyrus
	5.33	54	0	−26	Right middle temporal gyrus, anterior division
	4.36	−6	62	34	Left superior frontal gyrus
	4.14	−6	−58	6	Left precuneus cortex
	3.98	34	−36	−2	Right hippocampus
UL > NL	6.1	44	44	14	Right middle frontal gyrus
	5.57	2	42	20	Right Paracingulate Gyrus
	4.94	50	−42	44	Right supramarginal gyrus, posterior division
	5.01	−48	−12	−6	Left superior temporal gyrus
	5.08	−32	−68	−34	Posterior lobe of left cerebellum
	5.18	4	−32	30	Right cingulate gyrus, posterior division
	4.32	16	−46	−32	Anterior lobe of right cerebellum
	4.3	−12	−74	28	Left cuneus/precuneus cortex
	3.7	−8	−54	−26	Anterior lobe of left cerebellum
	4.62	−28	−70	−60	Left cerebellum
	4.11	−46	−60	48	Left angular gyrus/inferior parietal lobule
	4.43	12	−68	40	Right precuneus cortex

Abbreviations—NL: Natural language condition; UL: unknown language condition; Contrasts between conditions are depicted by “>” meaning more than, i.e., NL > UL: areas in which the condition NL elicited a higher BOLD response than UL). MNI: Montreal Neurological Institute coordinate system.

## Data Availability

Due to the personal and sensitive nature of data collected from volunteers of this research, the authors require to be contacted in order to inform participants and provide necessary arrangements for data access according to Brazilian Data Protection Law. Data available via request.
